# Will We Do If We Can? Habitual Qualitative and Quantitative Physical Activity in Multi-Morbid, Older Persons with Cognitive Impairment

**DOI:** 10.3390/s20247208

**Published:** 2020-12-16

**Authors:** Bastian Abel, Martin Bongartz, Tobias Eckert, Phoebe Ullrich, Rainer Beurskens, Sabato Mellone, Jürgen M. Bauer, Sallie E. Lamb, Klaus Hauer

**Affiliations:** 1 Department of Geriatric Research, AGAPLESION Bethanien Hospital Heidelberg, Geriatric Center at the University of Heidelberg, 69126 Heidelberg, Germany; bastian.abel@bethanien-heidelberg.de (B.A.); bongartz@nar.uni-heidelberg.de (M.B.); tobias.eckert@kit.edu (T.E.); phoebe.koepp@bethanien-heidelberg.de (P.U.); rainer.beurskens@fh-mittelstand.de (R.B.); juergen.bauer@bethanien-heidelberg.de (J.M.B.); 2 Center for Geriatric Medicine, Heidelberg University, 69126 Heidelberg, Germany; 3 Network Aging Research (NAR), Heidelberg University, 69115 Heidelberg, Germany; 4 Department for Social and Health Sciences in Sport, Institute of Sports and Sports Science, Karlsruhe Institute of Technology, 76131 Karlsruhe, Germany; 5 Department of Health and Social Affairs, FHM Bielefeld, University of Applied Sciences, 33602 Bielefeld, Germany; 6 Department of Electrical, Electronic, and Information Engineering, University of Bologna, 40136 Bologna, Italy; sabato.mellone@unibo.it; 7 Institute of Health Research, University of Exeter, South Cloisters, St. Luke’s Campus, Exeter EX1 2LU, UK; s.e.lamb@exeter.ac.uk

**Keywords:** sensor-based, activity behavior, gait, turning, symmetry, regularity, qualitative, determinants

## Abstract

This study aimed to identify determinants of quantitative dimensions of physical activity (PA; duration, frequency, and intensity) in community-dwelling, multi-morbid, older persons with cognitive impairment (CI). In addition, qualitative and quantitative aspects of habitual PA have been described. Quantitative PA and qualitative gait characteristics while walking straight and while walking turns were documented by a validated, sensor-based activity monitor. Univariate and multiple linear regression analyses were performed to delineate associations of quantitative PA dimensions with qualitative characteristics of gait performance and further potential influencing factors (motor capacity measures, demographic, and health-related parameters). In 94 multi-morbid, older adults (82.3 ± 5.9 years) with CI (Mini-Mental State Examination score: 23.3 ± 2.4), analyses of quantitative and qualitative PA documented highly inactive behavior (89.6% inactivity) and a high incidence of gait deficits, respectively. The multiple regression models (adjusted *R*^2^ = 0.395–0.679, all *p* < 0.001) identified specific qualitative gait characteristics as independent determinants for all quantitative PA dimensions, whereas motor capacity was an independent determinant only for the PA dimension duration. Demographic and health-related parameters were not identified as independent determinants. High associations between innovative, qualitative, and established, quantitative PA performances may suggest gait quality as a potential target to increase quantity of PA in multi-morbid, older persons.

## 1. Introduction

Decreased motor capacity, defined as maximal level of functioning under standardized conditions [1], as well as decreased motor performance of physical activity (PA), describing habitual behavior in someone’s actual environment [1], are common in older persons [2,3] and associated with various negative health outcomes, such as motor impairments, falls, affected psycho–social status, cardiovascular diseases, or mortality [4,5,6]. In addition to disease- or impairment-related conditions, such as stroke, orthopedic disorders, or pain [7], cognitive impairment (CI) stands out as it has been associated with poor qualitative and quantitative measures of gait [8] and a low volume of PA performance [9] with negative consequences on psychological status as well as social deprivation [10,11].

Although there is still no gold standard in sensor-based activity monitoring [12], technical developments allow to overcome previous limitations in questionnaire-based assessments of PA performance and to document the volume of PA performance by established, quantitative dimensions, such as duration, frequency, or intensity, as stated by leading medical associations [13]. Among all PA performance measures, walking has been considered as the key habitual motor activity that is most often reported but mainly assessed by established, quantitative parameters such as number of steps or duration of walking. These parameters have been increasingly complemented by innovative, qualitative measures, including characteristics of straight walking (e.g., symmetry, regularity, or variability of gait) [14] and turning while walking (e.g., duration, angle, or velocity of turns) [15], which enable qualitative analyses of walking as a key feature of habitual PA performance. Such qualitative characteristics allow to better understand the mechanisms of motor failure, such as falls [16], and have been successfully implemented to predict falls in older persons [14,15]. These qualitative variables of PA performance are also related to mild CI [17] and neurodegenerative disorders such as Parkinson’s disease [18,19] and multiple sclerosis [20], as documented by cross-sectional analyses comparing middle-aged or older persons affected by these diseases with healthy controls. Furthermore, qualitative measures of walking capacity under laboratory conditions have also shown associations with the psychological status (e.g., depression, fear of falling) [21] and activity restrictions [22], thus documenting their sensitivity for psychological influences and activity behavior.

Turning while walking stands out as a more demanding movement for older persons, compared to straight walking [23,24], and is required in many daily activities [25]. It reflects a high risk situation for serious falls that my lead to hip fractures [26] and has therefore been incorporated in established clinical tests, such as the Timed “Up & Go” Test. For this test, good predictive validity for adverse health outcomes, fear of falling, and future falls has been documented [27,28,29]. Similar to the detailed analysis of straight walking, the quantitative turning capacity has recently been amended by qualitative characteristics such as turning velocity or turning angle, enabling detailed insights into turning while walking [15,30]. While such qualitative capacity measures have improved the understanding of habitual activity, namely gait-related performance, results of predictive validity are heterogeneous when using exclusively laboratory-based measures [31,32,33]. Gait characteristics assessed under highly standardized, laboratory-based conditions (walking capacity) differ substantially from gait characteristics measured during non-standardized, habitual PA (walking performance) [34], which may be affected by frequent distractions with potential negative consequences on habitual PA behavior. However, the assessment of qualitative characteristics of PA performance still remains a methodological challenge, especially in multi-morbid, older persons with activity clusters and gait performances that are hard to detect and classify [35]. While qualitative examinations of gait under habitual conditions are getting more and more attention [14,15,30,36], sensor-based assessment methods were predominantly validated in laboratory settings [19,37] with very restricted use in the real-life assessment of frail, older persons [35,38].

The potential of innovative, qualitative characteristics of PA performance have so far mainly been used in discriminative studies to describe motor differences between healthy, high functioning persons as compared to impaired populations [14,15,18,19,30,36]. These studies on specific qualitative characteristics of gait performance in habitual settings of older adults focused on either, variables of turning [15,30], or parameters of straight walking [14,36], but did not use the whole range of parameters. While such discriminative, observational studies found significantly decreased qualitative motor performance (e.g., lower gait symmetry or lower turning velocity) in persons with falls [14,15,30,36] as well as in those with Parkinson’s disease [18,19], it is interesting that in most of these studies, the quantity of PA performance did not differ as compared to healthy study groups [15,18,30,36]. These findings support the assumption that qualitative characteristics of PA performance are particularly important to complement the more established quantitative parameters.

To represent specific disease-related symptoms, such qualitative measures are of particular interest for specific patient groups such as persons with CI. Especially older adults with CI have a reduced gait quality, which is not only a risk factor for falls but also a predictor for further cognitive decline [39,40]. However, research on qualitative characteristics of habitual PA performance in older persons with CI is scarce. Only three studies with cross-sectional analyses have been identified that considered older persons with CI in subgroups, comparing merely few, individual qualitative characteristics such as variability or regularity of gait performance between older adults with and without CI [17,41,42]. These discriminative studies showed deteriorations in the qualitative motor performance of younger old adults with CI [42] as well as of older persons with fairly preserved motor capacity and CI [17,41] in comparison with age-matched controls.

In previous studies that aimed to identify determinants of PA performance, parameters of socio-demographic, medical, psychological, cognitive, or functional status were used and indicated low to moderate associations with quantitative parameters of habitual PA [43,44,45,46,47,48]. A restricted number of studies that have analyzed associations of motor capacity, determined by standardized protocols, with quantitative motor performance, documenting habitual activity behavior, showed moderate to high associations [38,49]. However, as such capacity measures deviate substantially from habitual PA performance, an open research question remains, whether qualitative motor performance during habitual activity may best determine quantitative motor performance during habitual activity.

To the best of our knowledge, a research approach to identify determinants of quantitative PA performance with focus on innovative, qualitative characteristics of gait performance in multi-morbid and older persons with motor and cognitive impairment has not been undertaken so far. Based on low levels of PA performance in older persons with CI [9], the development of specific interventions to increase PA performance and thereby minimize the likelihood of negative health outcomes in this high-risk population is a persistent goal of current geriatric research. In order to implement most effective interventions for these multi-morbid, older adults, it is essential to understand the determinants of quantitative PA performance including duration, frequency, and intensity. The association between the quality of habitual activity and the quantity of activity behavior (will we do if we can?) will be most relevant with direct consequences on the development of future training programs.

The main aim of this study therefore was to identify potential determinants of established, quantitative parameters of habitual PA performance by using innovative, qualitative characteristics of gait performance, in addition to established variables such as demographic and health-related parameters and measures of motor capacity, in multi-morbid, older persons with mild-to-moderate CI. A further aim was to describe the innovative, qualitative, and established, quantitative variables of habitual PA performance. Based on results of previous research [38], we hypothesized low to moderate associations of demographic and health-related variables as well as moderate to large associations of motor capacity and qualitative gait variables with the quantitative parameters of PA performance.

## 2. Materials and Methods

### 2.1. Study Design and Study Population

The present study is a cross-sectional observational study which used pre-intervention data from a randomized controlled trial (RCT) on effects of home-based training following inpatient rehabilitation [50]. The RCT was registered (ISRCTN82378327), ethically approved (Medical Faculty of the University of Heidelberg: S-252/2015) and performed according to the Declaration of Helsinki. Between September 2015 and April 2017, older participants (age ≥ 65 years) with mild-to-moderate CI (Mini-Mental State Examination (MMSE) score of 17–26 points; [51]) were consecutively recruited during geriatric rehabilitation. Further inclusion criteria were ability to walk at least 4 m without walking aid, community-dwelling, no delirium, no terminal disease, adequate language ability, residence within 30 km of the study site, and written informed consent.

### 2.2. Measurements

#### 2.2.1. Demographic and Health-Related Variables

Age, gender, and number of medications were documented from patient charts at discharge from inpatient rehabilitation. Further outcome measures including fall-related self-efficacy (Falls Efficacy Scale-International short version; FES-I short [52]), fall-related avoidance behavior (Fear of Falling Avoidance Behavior Questionnaire; FFABQ [53]), depressive symptoms (Geriatric Depression Scale short form; GDS-SF [54]), and care grade (yes vs. no) were documented by trained assessors in standardized interviews at the participants’ home before intervention. Care grade defines benefits of the statutory German long-term care insurance associated to individual, comprehensive care needs as described elsewhere [55,56].

#### 2.2.2. Motor Capacity

The Short Physical Performance Battery (SPPB; including subtests of balance, gait, and chair rise capacity [57]), habitual gait speed (based on SPPB), and Timed “Up & Go” Test (TUG [58]) were also assessed at the participants’ home under strictly standardized conditions.

#### 2.2.3. Physical Activity Performance

In the participants’ home environment, habitual PA performance was measured for 48 h with the uSense activity monitor (attached to participants’ lower back using adhesive bands), a non-commercial activity monitor developed in a large EU-funded project (FARSEEING, FP7/2007–2013, Grant No. 288940), allowing ambulatory, long-term assessment of PA. The assessments of PA performance were considered valid if they included data on at least 48 h of activity measurement. Concurrent validity of the uSense have been proved under standardized conditions by simultaneous annotated video recording during scripted and unscripted activities of frail, older persons, showing an agreement of 95.1% and 92.8%, respectively [59]. Good construct validity, verified for qualitative variables of gait performance and parameters of habitual PA, and excellent test–retest reliability (intraclass correlation coefficients for two consecutive days: 0.68–0.97) for most of these parameters have been shown under habitual conditions in multi-morbid, older persons with motor and cognitive impairment [38], representing the target population of the present study. This small-scaled monitor (42 × 10 × 68 mm, 36 g) includes a 9-axis inertial platform with three different types of motion sensors (accelerometer, gyroscope, and magnetometer) to generate a large variety of parameters of PA performance. These parameters have been distinguished into quantitative variables that describe established parameters of PA and gait performance (activity counts; number of steps; duration of lying, walking, inactive behavior (without lying), and active behavior (without walking); number of lying episodes, walking episodes, inactive episodes (without lying episodes), and active episodes (without walking episodes); mean metabolic equivalent of task (METs); mean METs per walking episodes; cadence; and step duration) and innovative, qualitative variables that describe characteristics of gait during straight walking (variability of step duration; anteroposterior (AP), mediolateral (ML), and vertical (V) step regularity; Phase Coordination Index; AP, ML, and V harmonic ratio) as well as turning (turning duration, turning angle, turning velocity, and number of turns). Step regularity has been defined as an inter-step autocorrelation coefficient in the AP, ML, and V planes, where a value of 1.0 indicates perfect regularity [60]. Phase Coordination Index, a measure of gait coordination/symmetry expressed in percent, describes the ability to coordinate bilateral sequences of steps within a stride [61], whereby a Phase Coordination Index of 0% reflects perfect left-right coordination [62]. Harmonic ratios quantify the step-to-step symmetry in AP, ML, and V directions, with higher values indicating greater gait symmetry [63]. Raw data was sampled at a frequency of 100 Hz, stored on internal storage, and analyzed offline using MATLAB (R2016a, The MathWorks Inc., Natick, MA, USA). Data processing and detailed definitions of the established parameters of PA performance are described elsewhere [38,64].

### 2.3. Statistical Analysis

Descriptive data are presented as means and standard deviations, medians and interquartile ranges (IQR), or numbers and percentages. Independent-samples *t*-tests and *χ*^2^-tests were used for the comparison of participant characteristics between persons with valid and invalid uSense measurements.

Based on a scientific statement of the American Heart Association [13], characterizing the volume of PA performance as a product of the quantitative dimensions duration, frequency, and intensity of PA performance in a given time frame, univariate linear regression analyses were performed to delineate potential determinants of these three dimensions. Since walking was identified as key aspect and essential focus of PA performance, the dependent variables of the univariate regressions consisted of the following established and quantitative variables of walking performance: duration of walking (minutes), frequency of walking (number of episodes), and intensity of walking (METs). To allow a more comprehensive analysis, we included an additional dependent quantitative variable, which was not directly derived from walking performance (the average total intensity (METs)), representing the total PA.

The independent variables were classified into following seven different domains, comprising established, quantitative motor, and non-motor parameters (domains 1–3), identified as potential determinants in previous comparable studies [4,43,44,45,46,47,65,66], and innovative, qualitative parameters of gait performance (domains 4–7): (1) demographic variables (age, gender), (2) health-related variables (number of medication, care grade, MMSE, GDS-SF, FES-I short, FFABQ), (3) motor capacity (SPPB, habitual gait speed, TUG), (4) variability of straight walking (coefficient of variation (CV) of step duration), (5) regularity of straight walking (AP, ML, and V step regularity), (6) symmetry of straight walking (Phase Coordination Index and AP, ML, and V harmonic ratio), and (7) qualitative gait parameters of turning while walking (turning duration, turning angle, mean turning velocity, peak turning velocity). The duration, angle, and velocity of turning were considered as qualitative measures since they document the commonly slower, smaller, and simplified movement strategies used by older persons to maintain their balance and compensate for loss of coordination [67,68,69].

Subsequently, four multiple linear regression models (stepwise forward, *p* ≤ 0.05 to enter) were performed to identify independent determinants of the different quantitative dimensions of walking (duration, frequency, and intensity) as well as the total PA performance (average total intensity). Only the independent variables with the highest, significant regression coefficient within each of the seven different aforementioned domains of the univariate analyses were included in the respective multiple linear regression models.

Potential multicollinearity of independent variables (*r* > 0.7 between independent variables, tolerance value < 0.2, and variance inflation factor (VIF) > 10 [70]) and the compliance of further assumptions for multiple linear regression analyses were considered (homoscedasticity and normality of residuals [71], and autocorrelation (Durbin-Watson-Test).

The multiple regression models were described by the adjusted coefficient of determination *R^2^* and influences of variables are given as unstandardized (Beta) and standardized (*ß*) regression coefficients. A two-sided *p*-value ≤ 0.05 indicated statistical significance. Data analyses were performed using SPSS Statistics 25 (IBM, Armonk, NY, USA).

## 3. Results

### 3.1. Descriptive Characteristics

The study sample included 110 multi-morbid (number of medications: 9.6 ± 3.5), older (82.3 ± 5.9 years) persons, discharged from ward-based geriatric rehabilitation, with mild-to-moderate CI (MMSE score: 23.3 ± 2.4 points), advanced motor impairment (SPPB score: 5.2 ± 2.3 points), and moderate concerns about falling (FES-I short: 12.3 ± 4.3 points). Sixteen sensor-based assessments of PA performance were invalid due to technical failure (*n* = 1), refusal to wear the activity monitor (*n* = 3), and premature removal of the uSense by participants (*n* = 12). No significant differences were found between characteristics of participants with valid versus invalid measurements (Table 1).

### 3.2. Physical Activity Performance

In order to describe the PA of the *n* = 94 included study participants with successful 48 h sensor-based measurements in more detail, PA performances have been classified into quantitative and qualitative parameters.

#### 3.2.1. Quantitative Parameters of Physical Activity Performance

During the 48 h activity monitoring, the established, quantitative parameters revealed a predominantly sedentary behavior during the day (21.5 ± 1.2 h (89.6%) inactive vs. 2.5 ± 1.2 h (10.4%) active), distinctive for multi-morbid, older persons with CI. The significantly shorter duration of walking, compared to lying per day (61 ± 47 min vs. 597 ± 117 min, *p* < 0.001), and the considerably higher number of walking episodes, compared to lying episodes per day (317 ± 226 episodes vs. 33 (median, IQR: 19–58) episodes, *p* < 0.001), are based on the mainly very short bouts of walking and longer bouts of lying. An average cadence of 74 ± 7 steps per minute and average step duration of 0.72 s (median, IQR: 0.68–0.79) implied slow habitual walking, also typical for the frail study population. Further details on the description of the quantitative parameters of PA performance are shown in Table 2.

#### 3.2.2. Qualitative Parameters of Gait during Physical Activity Performance

The qualitative gait parameters of straight walking showed a high CV of step duration, low inter-step correlation coefficients, a high Phase Coordination Index, and low harmonic ratios (Table 2).

**Figure 1:**
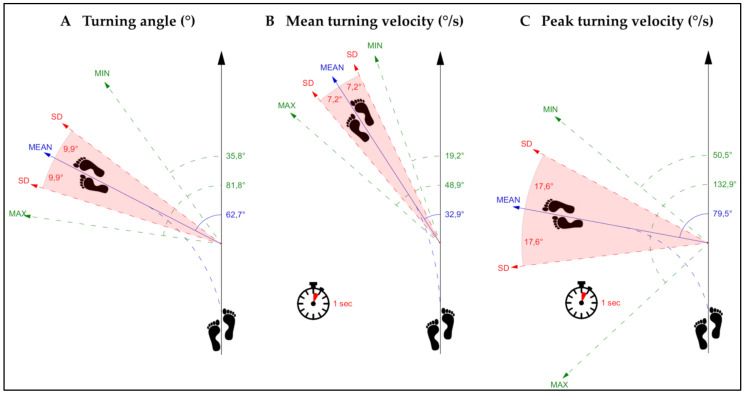
This figure presents the average values of (**A**) turning angle, (**B**) mean turning velocity, and (**C**) peak turning velocity. SD = standard deviation, MIN = minimum, MAX = maximum, ° = degrees.

The investigation of innovative, qualitative gait characteristics of turning while walking revealed a cautious turning behavior with a prolonged average turning duration, a low average turning angle as well as a slow average mean and peak turning velocity (Table 2; Figure 1). The total number of turns per day during the 48 h PA performance assessment was 514 (median, IQR: 155–973) and the average number of turns per walking episode was 2.1 ± 0.5.

### 3.3. Determinants of Physical Activity Performance

To identify determinants of established, quantitative parameters of PA performance, univariate regressions with multi-domain variables were used to detect potential determining variables which were included in the final multiple regression models.

#### 3.3.1. Univariate Regressions

Non-motor variables including demographic variables (age and gender) and health-related variables (e.g., number of medication and care grade as a surrogate marker of multi-morbidity and functional dependency, respectively, or cognitive status (MMSE)) showed moderate and mostly singular associations with the established, quantitative dimensions of PA performance. Only the FFABQ, documenting activity avoidance related to concerns about falling, was moderately associated with all of the quantitative dimensions of PA performance (*β*: |0.233–0.320|, *p*: 0.002–0.024). More detailed outcomes of the univariate analyses are given in Table 3.

In contrast, all variables of motor capacity showed moderate to high associations with each of the quantitative dimensions of PA performance (*β*: |0.347–0.580|, all *p*: ≤ 0.001; Table 3).

Innovative, qualitative characteristics related to variability, regularity, or symmetry measures of gait performance while walking straight revealed moderate associations with all quantitative parameters of walking and total PA performance; however these associations were heterogeneous. While six out of eight and five out of eight of the qualitative variables of straight walking were associated with the duration and frequency of PA performance, respectively, two out of eight were related to both of the intensity parameters (Table 3).

All qualitative parameters of gait performance while walking turns stood out with predominantly high associations to all quantitative parameters of PA performance (*β*: |0.299–0.787|, *p*: < 0.001–0.003; Table 3).

In general, the univariate regressions showed similar results across the established, quantitative dimensions of walking and total PA performance used as dependent variables in the regression models. Within dependent variables, results between duration and frequency and between walking intensity and overall intensity were most comparable.

#### 3.3.2. Multiple Regressions

None of the demographic or health-related measures remained as an independent determinant in any of the multiple regression models (Table 4, Table 5, Table 6 and Table 7).

Despite the moderate to high associations in the univariate regressions, the motor capacity measures did also not remain in the multiple regression models with the single exception of the SPPB as an independent determinant for the duration of walking (*β* = 0.250, *p* = 0.008; Table 4).

Among the innovative, qualitative characteristics of straight walking, only single measures remained in the multiple regression models. While the AP harmonic ratio was independently associated with the duration and frequency of walking (Models 1 and 2, Table 4 and Table 5), the ML and AP step regularity was independently associated only with the intensity of walking and total PA performance, respectively (Models 3 and 4, Table 6 and Table 7). The variability of step duration, the V step regularity, the Phase Coordination Index, the ML harmonic ratio, and the V harmonic ratio did either not remain in any of these multiple models or were not included as the univariate association was lower, compared to other variables within the same domain, or lacking.

With regard to the innovative, qualitative characteristics of walking turns, the mean turning velocity was an independent determinant for the duration and frequency of walking (Models 1 and 2) as well as the intensity of total PA performance (Model 4), whereas the peak turning velocity was an independent determinant for the intensity of walking (Model 3). As either the mean or peak turning velocity showed the highest univariate associations with the established parameters of PA performance within the domain of qualitative gait parameters of turning while walking, the average turning duration and average turning angle were not included in the multiple regression models.

The explained variance was relatively large in all models (adjusted *R^2^* = 0.395–0.679; all *p* < 0.001) given the limited number of determinants and the complexity of potential influences on PA behavior. No multicollinearity (all *r* < 0.7 between independent variables, minimal tolerance: 0.718, maximal VIF: 1.393) or autocorrelation (Durbin–Watson: 1.633–2.027) were present in any of the multiple regression models, indicating the independence of included variables.

## 4. Discussion

To the best of our knowledge, this cross-sectional analysis is the first showing that innovative, qualitative gait characteristics of straight walking and turning, documented in real-life settings, represent major determinants of established, quantitative dimensions of PA performance in multi-morbid, older persons with motor and cognitive impairment. Established demographic and health-related variables as well as motor capacity measures were not or only in a limited way associated with quantitative PA performance. In addition to the highly sedentary behavior, the high incidence of gait deficits under habitual conditions was documented by valid parameters of gait quality not analyzed in such detail in this vulnerable population before.

### 4.1. Physical Activity Performance

The sedentary behavior and low intensity of walking or total PA performance of multi-morbid, older adults with CI as documented by established parameters in the present study is consistent with findings from comparable samples [9,72] and typical for vulnerable, older persons. To complement such established parameters of PA performance with innovative, qualitative characteristics of gait performance is a novel approach that enables a more detailed insight into habitual physical behavior. These qualitative parameters of gait performance are of particular importance as a low quality of walking under everyday conditions is associated with several health-related aspects in older persons, such as mild CI [17], Parkinson’s disease [18,19], or a high risk of falling [14,15].

#### 4.1.1. Variability of Straight Walking

The variability of straight walking in the present study is high, but benchmarking is limited by missing comparable values for the CV of step duration from real-life settings in older adults with CI. When compared to a laboratory-based reliability study in older persons with CI [73], a substantially higher variability of step duration is visible in the present study. This divergence indicates the importance of separate qualitative measurements during daily activities, which, compared to highly standardized laboratory measurements, are affected by diverse external factors but represent the everyday life and relevant individual habitual activity behavior.

#### 4.1.2. Regularity of Straight Walking

With regard to the established definition of perfect step regularity, indicated by an inter-step autocorrelation coefficient of 1.0 [60], the step regularity of gait performance in the present study was low (0.32–0.44), no matter whether in AP, ML, or V direction. The step regularity in the present vulnerable, older study sample was lower as compared to the step regularity of habitual gait performance in younger and fitter old persons with and without CI [34], indicating negative effects of higher age, lower motor capacity, and CI.

#### 4.1.3. Symmetry of Straight Walking

Based on given definitions for the Phase Coordination Index (0% reflects perfect coordination) [61,62] and harmonic ratios (higher values indicate greater symmetry) [63], the left-right coordination (38.5 ± 5.0%) and step-to-step symmetry (1.06–1.43) of gait performance in the present study were low. As no results for the Phase Coordination Index from real-life settings were identified in older persons, laboratory-based studies in octogenarians with and without CI were used for comparison [74,75,76]. These laboratory studies rate much smaller values of the Phase Coordination Index (6.7–7.0%) as impaired gait coordination, which demonstrates the poor coordination of habitual gait as well as the impact of everyday life on the gait quality in the present vulnerable, older study sample.

The harmonic ratios in the present study were considerably smaller than in previous studies (1.82–2.25) that have examined associations of step-to-step symmetry of habitual gait performance with falls or time to falls in on average seven years younger persons with and without CI [14,77], again confirming effects of higher age and multiple impairments on the gait symmetry of the study sample as discussed above for step regularity.

#### 4.1.4. Turning while Walking

Although the mean turning duration in the present study was almost in line with findings from discriminative, observational studies in real-life settings in sixty-five-year-olds with Parkinson’s disease [19] or cognitively intact older adults with and without falls [15,30], the mean turning angle was approximately 30° smaller [15,19,30] and the mean turning velocity more than 10°/s slower [30], indicating qualitative compensation strategies for increased deficits in balance and coordination [68,69] in old age or CI.

In addition, the absolute number of turns in the present study was about 350 turns per day lower as compared to an age-matched peer group without CI, better turning performance, and better walking capacity [15]. The descriptive study results therefore suggest that quality and quantity of turning performance are sensitive indicators of motor restrictions in older persons with motor and cognitive impairment, such as the present study sample.

### 4.2. Determinants of Physical Activity Performance

While habitual PA performance has so far mainly been documented as established, quantitative dimensions in contexts of both scientific and public use, the qualitative characteristics of PA performance played a much lesser role due to methodological limitations for valid assessments in habitual settings. The potential determinants of quantitative PA performance were therefore restricted to established demographic and health-related variables, and parameters of motor capacity.

Accordingly, the main objective of the present study was to identify determinants of PA performance by using innovative, qualitative measures of habitual PA as compared to demographic and health-related variables, and parameters of motor capacity in multi-morbid older persons with CI, in which detailed gait and activity analyses are methodologically challenging but urgently needed.

#### 4.2.1. Univariate Regressions

To identify determinants of quantitative PA performance, a step-wise procedure was used. In a first step, variables from various domains were analyzed in univariate regression models.

- Demographic and health-related variables

As hypothesized, the demographic and health-related variables showed only moderate and singular relationships with the quantitative dimensions of PA performance, documenting lesser associations of generic domains or assessments that have been developed for clinical documentation rather than the prediction of PA. Study results are in line with previous studies, showing low to moderate negative univariate associations of advanced age, female gender, or various health-related variables with habitual PA performances such as overall intensity or activity counts [43,45]. These negative associations may indirectly relate to the lower motor capacity in persons with a poorer health status, higher age, or female gender [57,78], as a relevant restraint to be physically active (“do if we can”).

- Motor capacity

The significant associations of all motor capacity variables with all parameters of quantitative PA performance in the univariate analysis verified the hypothesis of a moderate to high association between motor capacity and quantity of PA performance in the study sample with impaired mobility. The study results confirm the moderate associations of various motor capacity measures (e.g., gait speed, SPPB and TUG score) with quantitative variables of PA performance (e.g., walking duration, walking frequency) in younger and fitter older adults [79,80].

Tests of motor capacity like the SPPB or TUG, as used in the present study, have been developed to investigate requirements and activities relevant to everyday life of older persons [57,58]. The moderate to high associations between motor capacity and quantity of PA in the present study confirm this methodological approach and the relevance of motor capacity as a key for habitual PA.

- Innovative, qualitative parameters of straight walking

In 15 out of 32 univariate analyses, qualitative gait parameters of straight walking showed significant associations with quantitative parameters of PA performance. This result partially confirms our hypothesis of moderate to high associations between the quality and quantity of performance and suggests that a better quality of habitual gait implies better motor skills as important prerequisite for a higher quantity of PA. Interestingly, these associations were mainly documented for the duration or frequency of PA performance and less for the intensity measures. This finding may be due to higher energy costs per unit time of intensive activity and the reduced ability to provide this energy in old age [81,82], indicating that high intensity activities are frequently restricted in multi-morbid, older persons such as the present study sample.

Only few studies investigated individual, qualitative variables of laboratory-based gait (qualitative motor capacity) as determinants of the quantity of PA performance in older adults [76,79,83,84], documenting heterogeneous results with generally limited associations: low or moderate negative univariate associations of gait variability with duration and frequency of moderate to vigorous PA or number of activity counts, respectively [83,84], low positive univariate associations of gait regularity with number of activity counts [84], and no or moderate positive univariate associations of gait symmetry with accelerometer-based or self-reported, questionnaire-based PA, respectively [76,79].

However, measures of qualitative capacity and qualitative performance may not be directly comparable [34]. While qualitative gait parameters of straight walking, including variability, regularity, and symmetry, under strictly standardized laboratory conditions predominantly document motor impairments (internal conditions), measures of qualitative performance may additionally cover effects of activity patterns and environmental or social interactions (external conditions) that are directly related to quality and quantity of PA. Qualitative measures of gait performance share the same context and setting with quantitative measures of habitual PA, allowing a high comparability by contrast with qualitative measures of gait capacity.

In addition to laboratory-based studies on associations between qualitative capacity measures and quantitative performance measures, studies on associations of qualitative gait performance while walking straight and the quantity of PA performance could not be identified, with the consequence that a direct benchmarking to the present results was not feasible.

- Innovative, qualitative parameters of turning while walking

All qualitative parameters of gait performance while walking turns were significantly and in most cases strongly associated with every quantitative dimension of PA performance in the present study. This result highlights the high proportion of turning during everyday indoor activities [25] and suggests that turning performance in vulnerable older persons represents a very sensitive indicator for the quality of gait in a complex and challenging movement, potentially leading to a higher quantity of PA performance (will we do if we can?).

The higher levels of PA performance in the present study were associated with lower turning duration as well as higher turning angle and velocity, which are usually prolonged or reduced, respectively, in older adults due to a loss of coordination as a compensation strategy to maintain balance [68,69]. Accordingly, walking turns represent challenging movements with high risk exposure for falling in multi-morbid, older persons with motor impairment [15,28,30].

While findings on laboratory-based turning measures (180°turning of the TUG) in older adults showed only a moderate positive univariate association of turning velocity with the number of activity counts [84], the predominantly high associations between qualitative measures of turning and quantitative dimensions of PA in the present study again indicate the higher relevance of habitual gait quality as compared to laboratory-based gait quality.

As studies on the relationship between qualitative measures of turning performance and quantitative measures of PA performance are lacking, benchmarking of the present findings with previous results was again not feasible.

#### 4.2.2. Multiple Regressions

In a final step, directly competing variables, each with the highest significant coefficients from the univariate regressions of the different domains, were analyzed in multiple regression models to ascertain independent determinants of the quantitative dimensions of habitual PA.

- Demographic and health-related variables

None of the demographic or health-related variables, included in the multiple regression models, remained as an independent determinant for the quantitative dimensions of PA performance. This result affirms the lesser suitability of demographic factors and health status for the determination of the quantity of PA, compared to motor variables, as already indicated by the results of the univariate analyses.

- Motor capacity

Although the measures of motor capacity document key motor functions [57,58] that are mandatory for habitual PA, the singular independent association between the SPPB and walking duration suggests that the measures of motor capacity are inferior in independently determining the quantity of PA performance, when directly compared to specific, selected qualitative parameters of PA performance. This singular or missing independent association between variables of motor capacity and quantitative PA performance may be explained by the generally different conditions of controlled laboratory research and field research [49,85], indicating a lesser degree of similarity between measures of motor capacity and quantitative dimensions of PA performance as compared to qualitative parameters of PA performance.

- Innovative, qualitative parameters of straight walking

The present study identified independent associations between specific qualitative variables of gait performance while walking straight (AP step-to-step symmetry and AP and ML step regularity) and all quantitative dimensions of PA performance, suggesting that qualitative characteristics of habitual gait are relevant independent determinants for the quantity of habitual PA.

The independent association of step-to-step symmetry with duration and frequency of walking may relate to typical deficits of older persons with CI, such as lower levels of PA performance [3] and a lower step-to-step symmetry [86], or the high association between step-to-step symmetry and walking balance [87,88], as a basic precondition of walking and thereby leading to a higher duration and frequency of walking.

In contrast, step regularity may be more important in the context of the PA dimension intensity, as it is indicated by the positive independent association of ML step regularity with intensity of walking and the negative independent association of AP step regularity with intensity of total PA performance. These contrary associations may be due to different reasons. One could be a frequent change in the participants’ progression speed that results in a lower AP step regularity but indicates a better ability to quickly adapt/change walking speed, while simultaneously the ML step regularity increases and reflects a better ML stability. This assumption suggests that a better qualitative motor performance enables a higher intensity of PA, which confirms our hypothesis of a strong association between qualitative and quantitative PA performance. Another reason could be the age-related decline in step regularity that only occurs in AP direction [89], assuming that a decreased AP gait control causes a higher energy expenditure, whereas a steady/good ML gait control in old age may facilitate a higher intensity of PA.

- Innovative, qualitative parameters of turning while walking

Either the mean or peak velocity of turning performance was the best independent determinant for all quantitative dimensions of PA performance in the present study, suggesting that qualitative parameters of habitual turning behavior represent superior determinants for the quantity of PA performance, compared to demographic and health-related variables, measures of motor capacity, and qualitative parameters of gait performance while walking straight.

The present findings are coherent since turning while walking represents a more challenging movement for older persons as compared to straight walking [23,24] that places high demands on coordination and balance [68,69], as prerequisites for activity, and accounts for up to almost fifty percent of daily indoor activities [25], which is typical for multi-morbid, older persons.

### 4.3. Strengths and Limitations

The present study is the first to use successfully validated methods [38] to document innovative, qualitative characteristics of habitual gait performance and to analyze the association of these parameters, besides non-motor variables and measures of motor capacity, with different quantitative dimensions of PA performance in multi-morbid, older adults with CI.

The inconsistent usage of technical terms in the literature (e.g., capacity vs. performance or performance vs. activity behavior, including qualitative as well as quantitative aspects) and the large variety of partially not validated sensor technologies, assessment methods, and outcome characteristics, due to a rapid technical development in the field of motion analysis in recent years, are limiting the benchmarking of the present findings and the comparability between different studies.

## 5. Conclusions

The present study results identified specific qualitative variables of gait performance, in particular while walking turns, as main determinants for the quantitative dimensions duration, frequency, and intensity of habitual PA (will we do if we can?) in multi-morbid, older persons with generally low gait quality and PA levels. Results indicate that the quality of motor performance may be superior to determine the quantity of motor performance as compared to established measures of motor capacity or demographic and health-related variables.

As qualitative measures of habitual performance are associated with adverse events such as falls [14,15,30], intervention programs with the focus to improve the quality of such performances may not only represent a key to increase the duration, frequency, and intensity of habitual PA but also reduce the risk of falling and thus counteracting a higher risk exposure due to achieved higher PA levels.

## Figures and Tables

**Table 1:** Participant characteristics and comparison of persons with valid and invalid measurements of physical activity performance.

Characteristics	Total Sample (*N* = 110)	Group of Persons with Valid Measurements (*n* = 94)	Group of Persons with Invalid Measurements (*n* = 16)	*p*
**Demographic variables**				
Age (years), mean ± SD	82.3 ± 5.9	82.4 ± 6.0	81.9 ± 5.2	0.757 *
Gender (women), number (%)	84 (76.4)	69 (73.4)	15 (93.8)	0.077 ^
**Health-related variables**				
Medication (number), mean ± SD	9.6 ± 3.5	9.6 ± 3.6	9.8 ± 3.3	0.811 *
Care grade (yes), number (%)	51 (46.4)	47 (50.0)	4 (25.0)	0.064 ^
MMSE (score), mean ± SD	23.3 ± 2.4	23.3 ± 2.4	23.7 ± 2.2	0.540 *
GDS-SF (score), mean ± SD	5.3 ± 3.0	5.3 ± 3.1	5.6 ± 2.7	0.720 *
FES-I short (score), mean ± SD	12.3 ± 4.3	12.0 ± 4.2	13.8 ± 4.6	0.113 *
FFABQ (score), mean ± SD	18.5 ± 12.6	17.9 ± 12.9	21.9 ± 10.4	0.242 *
**Motor capacity**				
SPPB (score), mean ± SD	5.2 ± 2.3	5.2 ± 2.3	5.3 ± 1.8	0.938 *
Habitual gait speed (meter/second), mean ± SD	0.46 ± 0.19	0.46 ± 0.20	0.43 ± 0.17	0.482 *
TUG (seconds), mean ± SD	24.4 ± 14.1	24.8 ± 15.0	22.3 ±6.9	0.296 *

This table presents descriptive variables of the total study sample and subgroups according to valid vs. invalid activity measurements. SD = Standard Deviation, MMSE = Mini Mental State Examination (0–30 pts., higher score indicates better cognitive status), GDS-SF = Geriatric Depression Scale-Short Form (0–15 pts., higher score indicates more depressive symptoms), FES-I short = Falls Efficacy Scale International-short version (6–24 pts., higher score indicates higher concerns about falling), FFABQ = Fear of Falling Avoidance Behavior Questionnaire (0–56 pts., higher score indicates greater activity avoidance due to fear of falling), SPPB = Short Physical Performance Battery (0–12 pts., higher score indicates better motor capacity), TUG = Timed “Up & Go” test (lower test time indicates better motor capacity). *p*-values as tested by * independent-samples *t*-test and ^ chi-square test are given for the comparison between subgroups with valid vs. invalid activity measurements.

**Table 2:** Physical activity performance of the 48 h measurement.

Variables	*n* = 94
**Established, quantitative parameters of physical activity performance**	
Total activity per day (counts/minute), mean ± SD	4831 ± 2088
Total steps per day (number), median (IQR)	4686 (1764–6996)
**Duration**	
Lying duration per day (minutes), mean ± SD	597 ± 117
Walking duration per day (minutes), mean ± SD	61 ± 47
Inactive duration without lying duration per day (minutes), mean ± SD	694 ± 123
Active duration without walking duration per day (minutes), mean ± SD	88 ± 40
**Frequency**	
Lying episodes per day (number), median (IQR)	33 (19–58)
Walking episodes per day (number), mean ± SD	317 ± 226
Inactive episodes without lying episodes per day (number), mean ± SD	1668 ± 667
Active episodes without walking episodes per day (number), mean ± SD	1667 ± 701
**Intensity**	
Average intensity of total physical activity performance (METs), mean ± SD	1.6 ± 0.1
Average intensity during walking episodes (METs), mean ± SD	2.4 ± 0.3
**Innovative, qualitative parameters of gait performance while walking straight or turns**	
**Variability of straight walking**	
Average CV of step duration (%), mean ± SD	29.6 ± 3.5
**Regularity of straight walking**	
Average AP step regularity (-), mean ± SD	0.37 ± 0.06
Average ML step regularity (-), mean ± SD	0.44 ± 0.10
Average V step regularity (-), mean ± SD	0.32 ± 0.07
**Symmetry of straight walking**	
Average PCI (%), mean ± SD	38.5 ± 5.0
Average AP harmonic ratio (-), mean ± SD	1.06 ± 0.09
Average ML harmonic ratio (-), mean ± SD	1.43 ± 0.17
Average V harmonic ratio (-), mean ± SD	1.15 ± 0.09
**Turning while walking**	
Average turning duration (seconds), mean ± SD	2.13 ± 0.50
Average turning angle (°), mean ± SD	62.7 ± 9.9
Average mean turning velocity (°/second), mean ± SD	32.9 ± 7.2
Average peak turning velocity (°/second), mean ± SD	79.5 ± 17.6

This table presents quantitative and qualitative parameters of physical activity performance. SD = Standard Deviation, IQR = Interquartile Range, MET = Metabolic Equivalent of Task, CV = coefficient of variation, AP = anteroposterior, ML = mediolateral, V = vertical, PCI = Phase Coordination Index. ° = degrees. Inactive duration/episodes: METs ≤ 1.5. Active duration/episodes: METs > 1.5.

**Table 3:** Univariate associations of multi-domain variables with quantitative dimensions of walking and total physical activity.

	Walking Performance						Total Performance	
	Duration (Minutes)		Frequency (Number of Episodes)		Intensity (METs)		Intensity (METs)	
Independent Variables	*β*	*p*	*β*	*p*	*β*	*p*	*β*	*p*
**Established, quantitative parameters**								
**Demographic variables**								
Age (years)	−0.209	0.043 *	−0.157	0.130	−0.305	0.003 *	−0.274	0.008 *
Gender (dummy; 0 = women, 1 = men)	0.176	0.089	0.043	0.680	0.197	0.058	0.209	**0.043**
**Health-related variables**								
Medication (number)	−0.219	**0.034**	−0.210	**0.042**	−0.131	0.206	−0.130	0.210
Care grade (dummy; 0 = no, 1 = yes)	−0.247	**0.016**	−0.238	**0.021**	−0.041	0.694	−0.155	0.137
MMSE (score)	0.132	0.205	0.129	0.214	0.209	**0.043**	0.214	**0.039**
GDS-SF (score)	−0.131	0.207	−0.109	0.298	0.006	0.954	−0.013	0.903
FES-I short (score)	−0.147	0.158	−0.147	0.158	−0.024	0.821	−0.090	0.387
FFABQ (score)	−0.320	0.002 *	−0.272	0.008 *	−0.233	0.024 *	−0.272	0.008 *
**Motor capacity**								
SPPB (score)	0.507	<0.001 *	0.506	**<0.001**	0.377	**<0.001**	0.535	**<0.001**
Habitual gait speed (meter/second)	0.491	**<0.001**	0.507	<0.001 *	0.419	<0.001 *	0.580	<0.001 *
TUG (seconds)	−0.384	**<0.001**	−0.347	**0.001**	−0.413	**<0.001**	−0.497	**<0.001**
**Innovative, qualitative parameters**								
**Variability of straight walking**								
CV of step duration (%)	−0.321	0.002 *	−0.234	0.023 *	−0.068	0.516	−0.094	0.367
**Regularity of straight walking**								
AP step regularity (-)	0.128	0.220	0.080	0.442	−0.264	**0.010**	−0.253	0.014 *
ML step regularity (-)	0.225	**0.029**	0.075	0.474	0.354	<0.001 *	0.174	0.094
V step regularity (-)	0.373	<0.001 *	0.283	0.006 *	0.140	0.178	0.170	0.102
**Symmetry of straight walking**								
PCI (%)	−0.305	**0.003**	−0.257	**0.012**	−0.066	0.525	−0.107	0.303
AP harmonic ratio (-)	0.448	<0.001 *	0.363	<0.001 *	0.130	0.213	0.209	0.043 *
ML harmonic ratio (-)	0.124	0.233	0.028	0.787	0.143	0.170	0.048	0.643
V harmonic ratio (-)	0.376	**<0.001**	0.277	**0.007**	0.132	0.206	0.155	0.137
**Turning while walking**								
Turning duration (seconds)	−0.299	**0.003**	−0.350	**0.001**	−0.498	**<0.001**	−0.524	**<0.001**
Turning angle (°)	0.527	**<0.001**	0.488	**<0.001**	0.454	**<0.001**	0.592	**<0.001**
Mean turning velocity (°/second)	0.559	<0.001 *	0.569	<0.001 *	0.651	**<0.001**	0.787	<0.001 *
Peak turning velocity (°/second)	0.315	**0.002**	0.302	**0.003**	0.694	<0.001 *	0.715	**<0.001**

This table presents the results of univariate regression analyses between variables of different domains with quantitative measures of walking performance and total performance. MMSE = Mini Mental State Examination, GDS-SF = Geriatric Depression Scale-Short Form, FES-I short = Falls Efficacy Scale International–short version, FFABQ = Fear of Falling Avoidance Behavior Questionnaire, SPPB = Short Physical Performance Battery, TUG = Timed “Up & Go” test, CV = coefficient of variation, AP = anteroposterior, ML = mediolateral, V = vertical, PCI = Phase Coordination Index. ° = degrees. *β* = standardized regression coefficient indicating low (<0.2), moderate (0.2–0.5), and high (>0.5) associations. *p*-values in bold face indicate significance (*p* ≤ 0.05). * included in the respective, subsequent multiple linear regression models.

**Table 4:** Independent determinants for the duration of walking performance.

Model 1: Walking Duration (Minutes)							
Independent Variables	Unstandardized Coefficients		Standardized Coefficients			Collinearity Statistics	
	Beta	SE	*β*	*t*	*p*	Tolerance	VIF
Mean turning velocity (°/second)	4.57	1.21	0.351	3.79	**<0.001**	0.718	1.393
AP harmonic ratio (-)	297.12	90.79	0.273	3.27	**0.002**	0.887	1.127
SPPB (score)	10.07	3.72	0.250	2.71	**0.008**	0.720	1.388

This table presents the results of a multiple regression analyses of variables from different domains with duration of walking performance as dependent variable. AP = anteroposterior, SPPB = Short Physical Performance Battery, Beta = unstandardized regression coefficient, SE = standard error, *β* = standardized regression coefficient, VIF = variance inflation factor. Adjusted coefficient of determination (*R^2^*) = 0.427. Durbin–Watson test for autocorrelation = 1.950.

**Table 5:** Independent determinants for the frequency of walking performance.

Model 2: Walking Frequency (Number of Episodes)							
Independent Variables	Unstandardized Coefficients		Standardized Coefficients			Collinearity Statistics	
	Beta	SE	*β*	*t*	*p*	Tolerance	VIF
Mean turning velocity (°/second)	33.86	5.41	0.535	6.25	**<0.001**	0.918	1.090
AP harmonic ratio (-)	1169.27	440.89	0.227	2.65	**0.009**	0.918	1.090

This table presents the results of a multiple regression analyses of variables from different domains with frequency of walking performance as dependent variable. AP = anteroposterior, Beta = unstandardized regression coefficient, SE = standard error, *β* = standardized regression coefficient, VIF = variance inflation factor. Adjusted coefficient of determination (*R^2^*) = 0.395. Durbin–Watson test for autocorrelation = 1.633.

**Table 6:** Independent determinants for the intensity of walking performance.

Model 3: Walking Intensity (METs)							
Independent Variables	Unstandardized Coefficients		Standardized Coefficients			Collinearity Statistics	
	Beta	SE	*β*	*t*	*p*	Tolerance	VIF
Peak turning velocity (°/second)	0.01	0.001	0.749	12.16	**<0.001**	1.000	1.000
ML step regularity (-)	0.96	0.18	0.329	5.33	**<0.001**	1.000	1.000

This table presents the results of a multiple regression analyses of variables from different domains with intensity of walking performance as dependent variable. ML = mediolateral, Beta = unstandardized regression coefficient, SE = standard error, *β* = standardized regression coefficient, VIF = variance inflation factor. Adjusted coefficient of determination (*R^2^*) = 0.658. Durbin–Watson test for autocorrelation = 2.027.

**Table 7:** Independent determinants for the intensity of the total performance of physical activity.

Model 4: Intensity of Total Physical Activity (METs)							
Independent Variables	Unstandardized Coefficients		Standardized Coefficients			Collinearity Statistics	
	Beta	SE	*β*	*t*	*p*	Tolerance	VIF
Mean turning velocity (°/second)	0.01	0.001	0.795	13.18	**<0.001**	0.981	1.019
AP step regularity (-)	−0.29	0.12	−0.150	−2.48	**0.015**	0.981	1.019

This table presents the results of a multiple regression analyses of variables from different domains with intensity of total physical activity performance as dependent variable. AP = anteroposterior, Beta = unstandardized regression coefficient, SE = standard error, *β* = standardized regression coefficient, VIF = variance inflation factor. Adjusted coefficient of determination (*R^2^*) = 0.679. Durbin–Watson test for autocorrelation = 1.666.
